# On the possible role of PIF in myofibrillar proteins mobilisation from skeletal muscles in the presence of non-malignant foci of growth in organism

**DOI:** 10.1038/sj.bjc.6604116

**Published:** 2007-11-20

**Authors:** E A Rapoport

**Affiliations:** 1 Klenovy bulv. 4-135, Moscow 115470, Russia

**Sir**,

In the article published in your journal, direct evidence was submitted for the first time that proteolysis inducing factor (PIF) expression proceeds not only in the tumour, but also in adjacent non-tumour tissues in gastro-oesophageal malignancy (Deans *et al*, 2006). This fact is in accordance with other ones, for example in tumour-bearing animals; the mitotic activity is also increased in extra-tumour tissues. As a result, liver growth is induced. The pattern of changes of metabolic activity of subcellular fractions of proteins in the liver of tumour-bearing animals correlates significantly with the pattern of changes in homonymous fractions in Zajdela ascites hepatoma (*R*=0.951) when compared with the intact liver, and in other series of experiments when liver growth was induced by partial hepatectomy, metabolic changes of the same protein fractions in regenerating liver also correlate strongly with those in ascites hepatoma (*R*=0.954) (Rapoport, 1964). One can suppose that during liver growth after partial hepatectomy, PIF could be also expressed in liver cells as in malignant cells. An indirect evidence in support of this assumption was presented in our work (Rapoport *et al*, 1996), namely: the content of myofibrillar proteins in white (fast–twitch) muscles is selectively reduced in the presence of regenerating liver in organism as it was found in tumour-bearing animals (Tisdale, 2006). In the latter, this phenomenon is induced by PIF, which activates the proteasome proteolytic system (Tisdale, 2006). Thus, the same mechanism of protein mobilisation from skeletal muscles may be involved in the presence of different foci of growth in organism through PIF production in them and its humoral transport to skeletal muscle system.
